# THE RELATIONSHIP BETWEEN LONELINESS AND PUBLIC TRANSPORTATION CARDS FOR OLDER PEOPLE

**DOI:** 10.1093/geroni/igae098.2577

**Published:** 2024-12-31

**Authors:** Murat Mercan, Oykum Yigit, Hulya Yurekli, Ilker Aslan

**Affiliations:** Yildiz Technical University, Istanbul, Istanbul, Turkey; Yildiz Technical University, Istanbul, Istanbul, Turkey; Yildiz Technical University, Istanbul, Istanbul, Turkey; Istanbul Metropolitan Municipality, Istanbul, Istanbul, Turkey

## Abstract

Active aging is critical to combating social isolation and loneliness. Scientific studies have found that even small increases in activity can reduce the risk of disease in the older people. One method that can be used to achieve these goals is providing discounted and free transportation cards for seniors. However, the number of studies measuring the effect of these cards on isolation and loneliness is very limited. This article aims to fill that gap. The paper consists of two parts. The first part is based on big data analysis. It investigates the daily transportation data provided by the Istanbul Metropolitan Municipality to examine how discounted (for individuals aged 60-65) and free (for those aged 65 and over) transportation cards affect the frequency of public transportation usage among individuals aged 50 and over between 2019 and 2022. The analysis, utilizing more than 400 million observations, holds tremendous original value. In the second part of the project, a representative survey is conducted among individuals aged 50 and over in Istanbul. This survey aims to measure the relationship between discounted and free transportation cards and social isolation and loneliness. Regression discontinuity, exploratory and confirmatory factor analyses, and logistic regression analysis methods will be employed for the analyses. The findings indicate an inverse relationship between loneliness and public transportation usage among the older people.

